# Individuals reciprocate negative actions revealing negative upstream reciprocity

**DOI:** 10.1371/journal.pone.0288019

**Published:** 2023-07-05

**Authors:** Ryohei Umetani, Hitoshi Yamamoto, Akira Goto, Isamu Okada, Eizo Akiyama

**Affiliations:** 1 Graduate School of Science and Technology, Degree Programs in Systems and Information Engineering, University of Tsukuba, Tsukuba, Ibaraki, Japan; 2 Faculty of Business Administration, Rissho University, Tokyo, Japan; 3 Meiji University School of Information and Communication, Tokyo, Japan; 4 Faculty of Business Administration, Soka University, Tokyo, Japan; 5 Faculty of Engineering, Information and Systems, University of Tsukuba, Tsukuba, Ibaraki, Japan; Teesside University, UNITED KINGDOM

## Abstract

Indirect reciprocity is widely recognized as a mechanism for explaining cooperation and can be divided into two sub-concepts: downstream and upstream reciprocity. Downstream reciprocity is supported by reputation; if someone sees you helping someone else, the person who sees this will think higher of you, and you will be more likely to be helped. Upstream reciprocity is helping someone because you are being helped by somebody else, which often happens in everyday life and experimental games. This paper focuses on the behavior of “take” and examines negative upstream reciprocity using an upstream reciprocity framework. The term “take” is defined as “to steal rather than give resources to others.” “If something is taken from you, do you take from others?” is an important extension for indirect reciprocity research; subsequently, this paper discusses experiments conducted on whether negative upstream reciprocity is chained and what causes it. The results demonstrated differences between positive and negative upstream reciprocity. In analyzing the data of nearly 600 participants to determine the extent to which negative upstream reciprocity is observed and the causes of negative upstream reciprocity, the study found that If individual A takes resources from individual B, then B is more likely to take resources from a third-party, individual C. Notably, some causes of positive upstream reciprocity were found to have no effect or the opposite effect on negative upstream reciprocity. The results also demonstrate that the first person to take can cause a chain reaction. This paper demonstrates the importance of the first person not taking from someone else and suggests the need to consider various behavioral options for future research on cooperation.

## Introduction

The evolution of reciprocal cooperation is crucial to the development of human society and is one of the biggest puzzles for science [1]. Cooperation is subject to social dilemmas, which, in terms of adaptation, should eventually weed out cooperators [2]. However, cooperative behaviors are evolving in many situations and have been widely studied in various disciplines, including social psychology [3, 4], economics [5, 6], physics [7, 8], and biology [9, 10].

**Fig 1:**
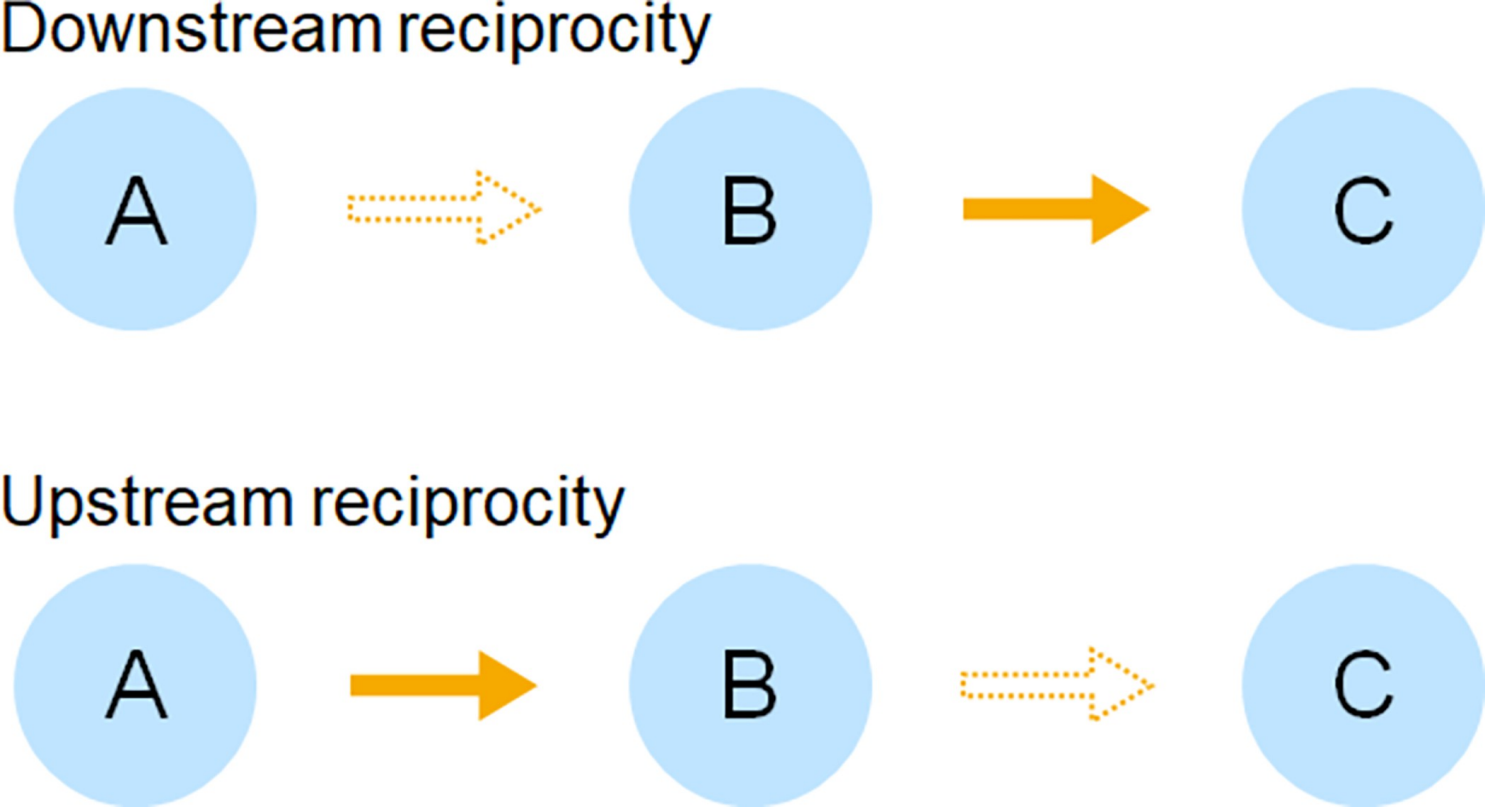
Difference between downstream and upstream reciprocity. This figure illustrates the difference between downstream and upstream reciprocity. In both cases, A is the person who cooperates with B, and C is the person B cooperates with. The arrow filled in orange is the one who cooperates first, and the dashed arrows are those who cooperate later. Downstream reciprocity is a mechanism whereby if you cooperate with someone, someone else who has been observing you will cooperate with you. On the one hand, upstream reciprocity means that if someone cooperates with me, I will also cooperate with someone else.

Evolutionary mechanisms of cooperation studies using evolutionary game theory have offered several possible solutions [11]. For example, cooperation is frequently observed between those in repeated interactional relationships [12, 13] and between blood relatives [14, 15]. An important evolutionary mechanism of cooperation is indirect reciprocity, which has been shown to explain the large-scale and flexible cooperation observed in humans [16–18]. Indirect reciprocity is not from the current party but from a third party [19]. This mechanism consists of two sub-concepts: downstream and upstream reciprocity [20]. Downstream reciprocity is a mechanism that supports a cooperative society based on reputation [21–25]. Upstream reciprocity occurs when a person who has received help helps a third person. This kind of cooperative behavior is often observed and is expressed in terms such as “pay it forward” [26]. Upstream reciprocity is promoted when cooperation generates gratitude and empathy [27–30], among other positive emotions [31, 32]. The difference between downstream and upstream reciprocity is illustrated in the figure (Fig 1).

Upstream reciprocity does not evolve by itself [29] but rather requires a certain number of situations, such as a small group size with fixed relationships [33, 34] or a network that maintains a community structure [35, 36]. Experimental studies have demonstrated that upstream reciprocal behavior is observed only as often as the probability of a coincidental chain of gullible individuals who only engage in prosocial behavior [37].

Reciprocity includes positive and negative reciprocity, characterized by responding to cooperation with cooperation and defection with defection, respectively [38–41]. In studies of negative reciprocity, those conducted in the direct reciprocity framework mainly target the reciprocation of “take” [42–44], whereas those in the upstream reciprocity framework target the chain of selfish inequitable distribution [45–48]. However, no study has focused on take chains in the framework of upstream reciprocity. Thus, our work is the first on negative upstream reciprocity targeting take chains.

The first objective of this study is to demonstrate the magnitudes of negative upstream reciprocity. Research on negative direct reciprocity has demonstrated that exchanges of not only give but also take occur between those in repeated reciprocal relationships [42] and tend to be more strongly chained and develop faster compared with positive direct reciprocity [43, 44]. Related studies on selfish inequitable distribution chains show that greedy distributions are more likely to be chained than generous distributions [48]. These previous studies suggest that the strength of the chain between positive and negative upstream reciprocity is asymmetric.

A previous study has demonstrated that greedy distributions are more strongly chained than generous distributions [48]. It was studied using the dictator game. The experiment was designed with four conditions, and participants were assigned to one of the conditions. The control condition behaved as the distributor in the normal dictator game, while the positive upstream condition first became the recipient in the dictator game and then took on the role of distributor. The starting amount for the dictator game in all conditions is $6. The positive upstream condition is divided into three conditions (generosity, equitable, and greedy), with the amount received upon first becoming a recipient being $6, $3, and $0, respectively. The experiment demonstrated that participants in the greedy condition made greedy distributions, participants in the equal condition made equal distributions, but participants in the generous condition made only equal distributions.

The second objective of this study is to demonstrate the effects of some factors explaining positive upstream reciprocity on negative upstream reciprocity. Positive upstream reciprocity may be an altruistic behavior that results from the belief in a just world (BJW) [49], which is the cognitive bias of believing that the world is fair [50–52]. It refers to the psychological tendency to believe that prosocial behavior has positive consequences, such as the acquisition of rewards and success unrelated to it, and that antisocial behavior has negative consequences, such as failure and punishment unrelated to it [53]. We examined whether BJW has a cooperation-promoting effect on negative upstream reciprocity. We also examined the effect on negative upstream reciprocity of the intentionality of cooperation, which has also been noted to promote cooperation in the framework of the ultimatum game and upstream reciprocity [54, 55].

A previous study demonstrating positive upstream reciprocity has conducted economic game experiments based on the trust game, and three roles (A, B, and C) are set up for the experiment [49]. In this experiment, a unique currency called G (gold) was used, which was then converted into JPY, and the participants were rewarded according to the G they held. The experiment begins with Role A being provided 400 G by the organizers as the source of funds. Role A then gives some of their G to Role B. The amount provided by Role A is doubled by the organizer, which goes to Role B, who provides Role C with any amount from the funds provided. In this case, the funds are not doubled, but the amount provided goes directly to Role C, who does not make decisions. The game is designed so that when both Roles A and B split half of their money, Roles A, B, and C have an equal gain of 200G each. The participants are told they will be randomly assigned to one of the three roles, but they are all assigned to Role B.

The experiment was designed with six conditions, and participants were assigned to one of the conditions. The first condition is the existence of Role A’s intention. There are two conditions: one is that the amount that Role A offers to the participant is determined by Role A’s intention, and the other is that the amount is determined mechanically, not by Role A’s intention. In addition, there are three conditions for the amount of money that Role A offers the participants (Greedy: 40G, Equality: 200G, Generosity: 400G). After the end of the economic game, the participants’ BJW was measured. The experiment demonstrates that those with higher BJW cooperated more in positive upstream reciprocity games.

## Method

We constructed a novel negative upstream reciprocity game using oTree [56]. The experiment was conducted on March 18, 2021. We recruited 594 participants using Yahoo! Crowdsourcing. Participation was by informed consent. We did not collect or access any personally identifiable information of the participants in this experiment. Excluding participants who stopped responding midway, 437 participants offered valid responses (69.3% male; *M*_age_ = 46.17 years). The respondents received economic rewards according to the money earned in the game.

This study was approved by the Rissho University Research Ethics Committee (application number 02–01). The participants were presented with a consent form for the experiment and accepted it before participating. In addition, participants were again presented with a document confirming their consent to participate immediately after the experiment began, and informed consent was provided by pressing a button confirming consent. The youngest participant in the experiment was 20 years old, and there were no minors.

We set three roles (A, B, and C) for the experiment. First, participants in all roles were provided with JPY 100 as a source of funds by the organizer. The order of decision-making was role A first, then role B; role C had no decision-making. Role A could take any amount of funds from Role B. Role B could do the same from Role C. The amount of money left in the hands of Role C was the indicator of cooperation. The participants were informed that they were randomly assigned to three roles. However, all of them were assigned to Role B. They were debriefed accordingly after the experiment.

**Fig 2:**
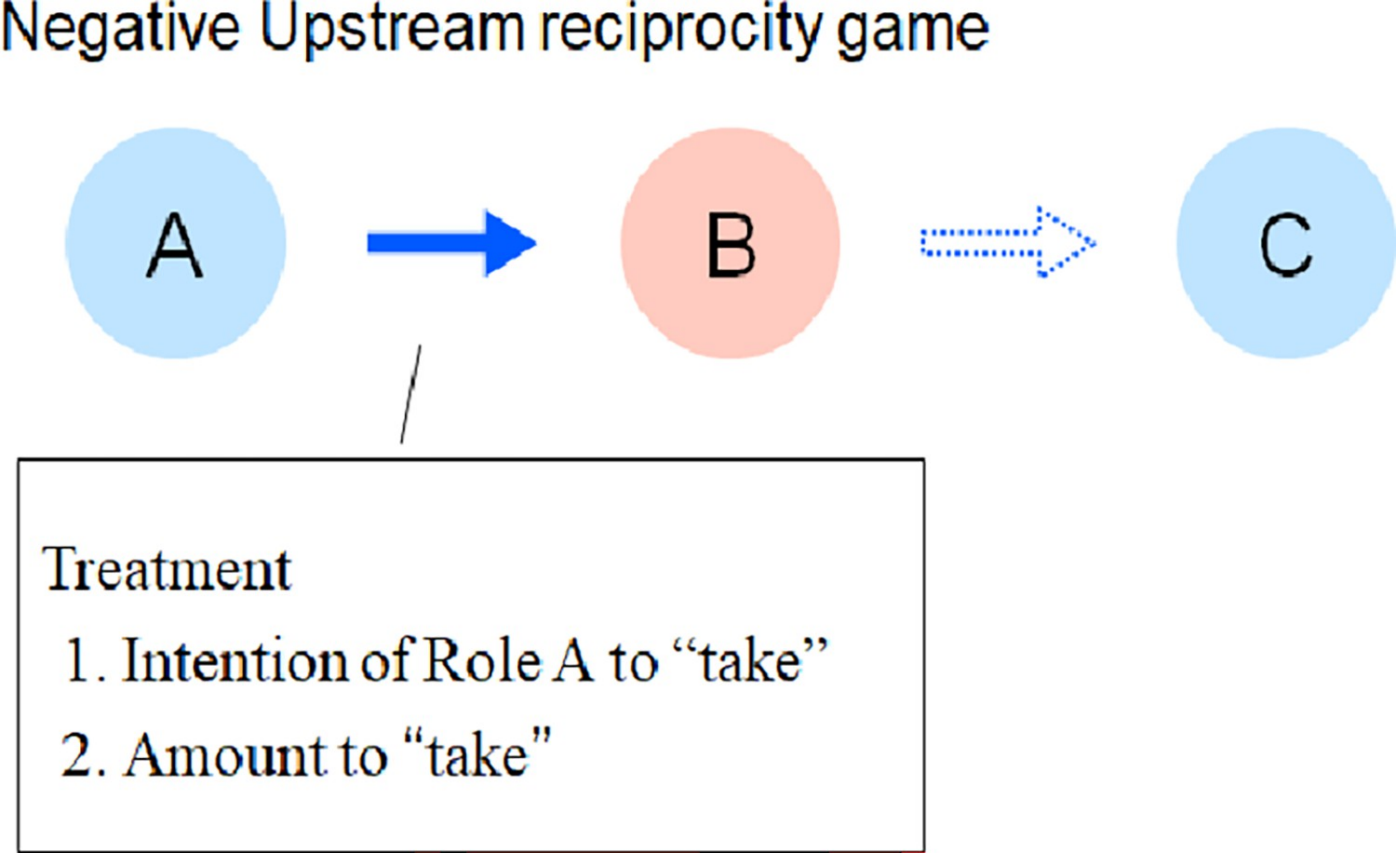
Experiment overview. An overview of the experiment and all participants are Role B in the figure. The color of the arrows in the previous figure differs from that in the figure above because the actions in the chain are different. The arrows in the previous figure represent cooperation, whereas the arrows in this figure represent take, so the colors of the arrows have been changed.

The experimental treatment consisted of six conditions: three conditions of the amount of money Role A takes from Role B (full, half, and none taken) and two conditions of the behavioral intention of Role A (Role A’s intention or unknown intention). The intentional condition means that Role A decides for itself how much to take from Role B. Unknown intentional condition means that Role A does not decide for itself how much to take from Role B but is forced to decide, like roulette. We used a between-subjects design. The following figure illustrates an overview of the experiment (Fig 2).

BJW was measured using a five-item scale (1 = *Disagree*, 5 = *Agree*) with four items each for the two factors of the belief in ultimate justice (BUJ) and belief in immanent justice (BIJ). The original factors were developed by Maes and Schmitt [57], but we used a Japanese version developed by Murayama and Miura [58]. In the experimental situation, the orientation toward resource allocation was considered to have a strong influence. Therefore, we measured social value orientation (SVO) as a controlling factor in the analysis [59].

## Results

**Fig 3:**
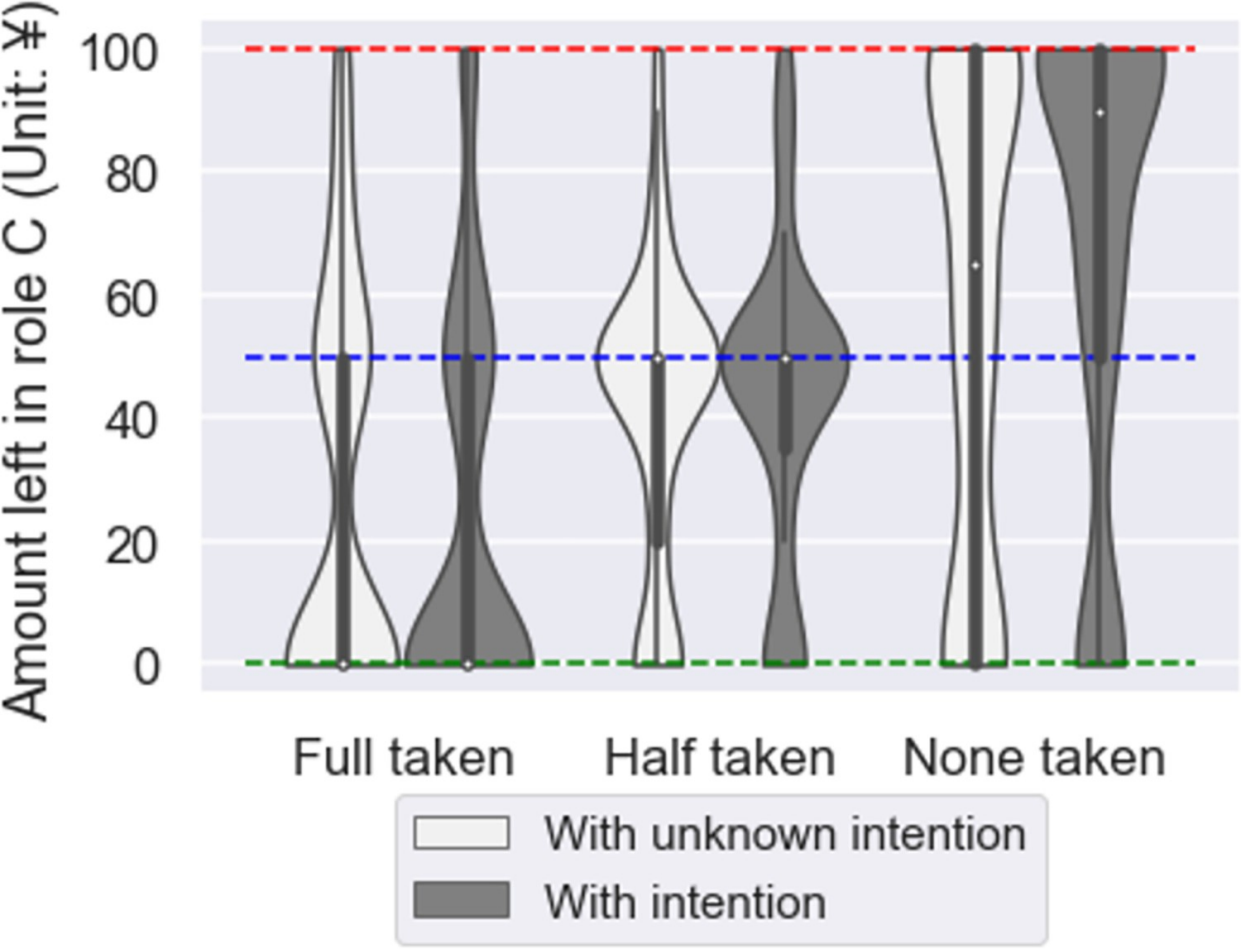
Violin plot of the amount of cooperation to third parties in each treatment. The vertical axis of this figure was truncated at 0 and 100 to exclude the data outside this range.

We conducted nonparametric tests for each factor to examine whether the amount taken by Role A and Role A’s intentions affected cooperation with the third party. We initially assumed parametric tests in the experimental design phase, but the normality tests of the distributions did not confirm normality. The Kolmogorov-Smirnov test did not confirm normality in the distribution under all treatments (all treatments *p <* .001). We conducted a Kruskal-Wallis test to examine the effect of the amount taken on cooperation with third parties. The results showed significant differences (χ^2^ = 80.764, *p* < .001). Next, a Mann-Whitney test was conducted to examine the effect of Role A intention on cooperation with third parties. We did not find any effect of Role A’s intention on cooperation with the third party (*p* = n.s.). This result suggests that if someone takes a resource, regardless of whether they intended to take it, they will take it back from a third party if it was taken (Fig 3). The broken lines in the figure represent Role A’s behavior toward the participant. The green line represents the “full taken” value by Role A from the participant, the blue line represents the “half taken” value, and the red line represents the “none taken” value.

Next, we examined the impact of BJW on negative upstream reciprocity by conducting an exploratory factor analysis (maximum likelihood method, Promax rotation) on eight items related to BJW. Subsequently, we extracted factors with the same structure as those from a previous study [58] and were adopted as BUJ (α = .94) and BIJ (α = .91).

We performed a multiple regression analysis with the amount of cooperation to third parties per amount taken condition as the dependent variable. The independent variables were BUJ, BIJ, behavioral intention in Role A, and an interaction term between the two BJW factors and behavioral intention. Age, sex, and SVO angle were set as control variables. The results demonstrated no main effect of BJW for either BUJ or BIJ. However, we observed a significant difference between behavioral intention and BUJ in the full taken conditions for the interaction (*p* = .003) (Table 1). A simple slope analysis demonstrated that differences were observed only in the intentionally taken condition, with those who had higher BUJ taking more from third parties when they were intentionally taken (β = -.303, *t* = -2.627, *p* < .05), but no difference in the amount of BUJ taking from third parties when there was unknown intentional taken (β = .183, *t* = 1.596, *p* = n.s.).

**Table 1:** Effect of the belief in a just world on negative upstream reciprocity.

	Full taken						Half taken						None taken					
	β	*SE*		β	*SE*		β	*SE*		β	*SE*		β	*SE*		β	*SE*	
Age	.017	0.254		.031	0.260		.000	0.205		.024	0.202		.112	0.290		.113	0.288	
Sex (0=male)	.128	5.559		.123	5.725		-.044	4.833		-.029	4.871		-.013	7.409		-.007	7.296	
SVO	.145	0.208+	+	.136	0.213		.179	0.173	*	.184	0.174	*	.399	0.251	**	.402	0.256	**
Intention	-.036	5.241		-.031	5.356		.049	4.240		.047	4.269		.138	6.149	+	.138	6.246	+
BUJ	-.060	2.636					.124	2.221					.049	3.522				
Int×BUJ	-.242	5.282	**				-.013	4.371					-.057	6.837				
BIJ				-.027	2.744					.010	2.219					.004	3.730	
Int×BIJ				-.147	5.428	+				.041	4.431					-.045	7.094	
*R^2^*	.097			.057			.053			.041			.213			.209		

** *p* < .01

* *p* < .05

+ *p* < .10

## Discussion

We examined negative upstream reciprocity. Our results demonstrated that if person A takes from B, the amount person B takes from person C is about the same as the amount taken by person A. The experiment revealed that negative upstream reciprocity has some different characteristics than the positive upstream reciprocity demonstrated in the previous study. The difference between the two in the amount of cooperation in the upstream reciprocity situation is expressed in the condition in which generous cooperation was given. In positive upstream reciprocity, generous distributions are not chained compared with fair or greedy distributions [48]. However, in none taken situations in negative upstream reciprocity, we found that significantly less was taken from third parties compared with other treatments. This difference may be due to differences in the behavior of give and take. Generally, taking behavior has a negative impression, which could have made the participants hesitate to maximize their self-interest. A previous study has pointed out that give and take have different meanings, even if they are economically equivalent [43]. Although this does not allow a perfect comparison, the amount of cooperation received by the subjects in this experiment was none, half, and full, which was similar to the previous study [48]. Therefore, a comparison with the previous study demonstrating the degree of positive upstream reciprocity linkage is meaningful.

The behavioral intention of Role A had no detectable effect on negative upstream reciprocal cooperation. The results also confirmed that negative and positive upstream reciprocity had different characteristics. In the taken situation under negative upstream reciprocity, a negative cycle of taking back from a third party occurred, even absent a behavioral intention on the part of the person who did the taking. This finding complements the results referred to in the hypothesis on equity with the world [60, 61]. The results of our experiment likewise complimented the finding that when a loss is inflicted on someone, the person who has suffered the loss will attempt to recover the gain, even from a third party, until the loss is fully restored [62], indicating that when they suffer damage, they will attempt to recover as much of one’s gain as possible regardless of whether the other person’s behavior was intentional or not.

A previous study that demonstrated that BJW promotes positive upstream reciprocity had a different experimental setting than the experiment conducted in this study [49]. However, the present experiment successfully set up a negative upstream reciprocity situation. Therefore, it is possible to compare the results on the impact of BJW on negative upstream reciprocity obtained in this study with the impact of BJW on positive upstream reciprocity in the previous study. This paragraph compares the results regarding the effects of BJW on negative upstream reciprocity with the effects of BJW on positive upstream reciprocity. Our study demonstrated that it had a partly negative effect on negative upstream reciprocity. This result again points to the difference in characteristics between positive and negative upstream reciprocity. When people cooperate in a positive upstream reciprocity situation, BJW reminds them of future losses, and the avoidance of which motivates them to cooperate with third parties [49]. Meanwhile, if taking is from others in a negative upstream reciprocity situation, BJW may provide the perception that taking from a third party to compensate for losses is justified.

**Fig 4:**
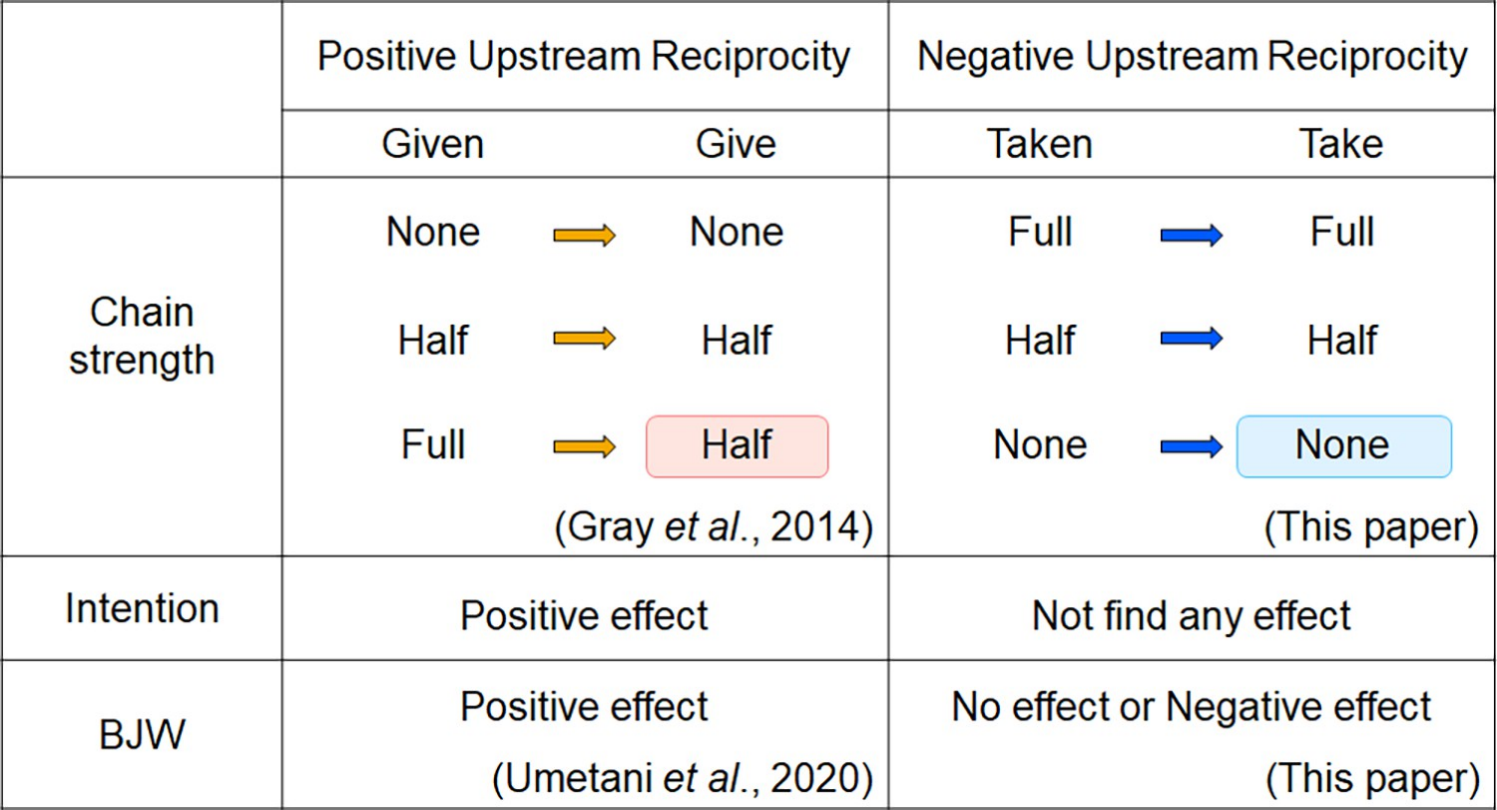
Comparison of positive and negative upstream reciprocity. In the figure, “Chain strength” compares the strength of the chain of positive and negative upstream reciprocal cooperation for each condition. The words and arrows in the same row have different colors for positive and negative because they have different behaviors. The orange arrow represents give, and the blue arrow represents take. The meanings of words are reversed in positive and negative because the actions are relative. For example, “Full” for positive upstream reciprocity means full give, and “Full” for negative upstream reciprocity means full take.

Our study is the first to focus on linkages of the negative upstream reciprocity. The results suggest that different behaviors of give and take may have different consequences, even when these are economically equivalent, consistent with previous findings [38, 41, 63, 64]. In terms of the amount of cooperation, positive upstream reciprocity only links about half of the cooperation, even with generous cooperation, whereas with negative upstream reciprocity, generous cooperation begets generous cooperation. Some factors positively affecting positive upstream reciprocity had no detectable effect on negative upstream reciprocity. Notably, the BJW that had a positive effect on positive upstream reciprocity had the exact opposite effect on negative upstream reciprocity, producing very different positive and negative reciprocity results. This result calls for the need to consider different behavioral options for studies on cooperation mechanisms. Fig 4 compares the present results with those on positive upstream reciprocity.

Humans develop through mutual cooperation but also engage in constant and deadly conflicts [65–67]. Despite numerous studies on the evolutionary mechanisms of cooperation, the research on the negative behavior of taking remains limited. This area is extremely important as it provides knowledge on avoiding the negative connections between several parties and achieving a cooperative society. As revealed in our experiment, the differences between positive and negative upstream reciprocity could indicate a simple solution to preserving a cooperative society: the chain of generous giving is weak and shrinking, whereas the same level of taking is chained in the framework of negative upstream reciprocity. In addition, behavioral intention does not influence negative upstream reciprocity when a take occurs. Thus, taking resources from others is likely to chain once it occurs. Efforts to avoid causing the taking are key to avoiding deadly conflicts.
